# Offline Reconstruction of Diffusion MRI Acquisitions for Comparison Between Complex PCA‐Based and AI‐Based Denoising

**DOI:** 10.1002/mrm.70336

**Published:** 2026-03-07

**Authors:** Francesco D'Antonio, Shaun Warrington, Jose‐Pedro Manzano‐Patron, Paul S. Morgan, Stamatios N. Sotiropoulos

**Affiliations:** ^1^ Sir Peter Mansfield Imaging Centre, School of Medicine The University of Nottingham Nottingham UK; ^2^ Mental Health and Clinical Neurosciences, School of Medicine The University of Nottingham Nottingham UK; ^3^ National Institute of Health and Care Research Nottingham Biomedical Research Centre, Nottingham University Hospitals Nottingham UK

**Keywords:** AIR‐recon DL, denoising, diffusion MRI, image reconstruction, MPPCA, NORDIC

## Abstract

**Purpose:**

Optimal diffusion MRI (dMRI) data for image denoising is often unavailable from scanner reconstruction. In this work, we make available an offline reconstruction pipeline for GE dMRI acquisitions, giving access to complex dMRI data. Furthermore, we compare the efficacy of GE HealthCare's AIR‐Recon DL (ARDL), a proprietary convolutional neural network‐based reconstruction and denoising approach, to patch‐based MPPCA_SVS_ and NORDIC denoising methods on high‐resolution dMRI data.

**Methods:**

We developed an end‐to‐end offline dMRI reconstruction pipeline for GE HealthCare acquisitions, augmenting the Orchestra software development kit, and validated its output against scanner reconstruction. We used it to compare MPPCA_SVS_, NORDIC, and ARDL denoising approaches, considering underlying metrics reflecting noise variance and bias, such as the signal profiles in highly anisotropic areas, and secondary downstream measurements, such as fiber orientation estimation and white matter tractography.

**Results:**

Our validated offline reconstruction supports various in‐plane/out‐of‐plane accelerations and partial Fourier reconstruction methods. Unlike scanner reconstruction, it provides access to complex dMRI data, enabling denoising in the complex domain, which demonstrated superior noise floor suppression compared with magnitude‐constrained denoising. PCA‐based denoising methods had improved spatial resolution, contrast‐to‐noise and more robust fiber orientation estimation compared with ARDL.

**Conclusion:**

We found significant gains in dMRI data quality when using the proposed offline reconstruction pipeline, allowing complex‐domain denoising to obtain high‐quality data at high spatial resolution and b‐value, using a wide‐bore scanner and a standard PGSE EPI sequence. MPPCA_SVS_ and NORDIC (4D PCA‐based) outperformed ARDL (2D) in terms of spatial resolution and reduction of noise variance.

## 1 Introduction

The value of denoising diffusion MRI (dMRI) data has been demonstrated in various scenarios, enhancing fidelity of dMRI‐derived microstructural estimates [1, 2, 3], increasing spatial resolution [2, 4], and improving consistency between scanners [5]. DMRI contrast is derived from signal attenuation, leading to low SNR, especially when increasing resolution or diffusion weighting. This results in low signals being rectified to the noise floor [6], leading to biases in parametric maps and downstream fiber modeling [3, 7, 8, 9]. To overcome these challenges, several denoising approaches have been developed using PCA‐based, low‐rank reduction of signals [5, 10, 11, 12, 13, 14, 15, 16, 17]. These methods exploit the spatial, angular and diffusion‐weighted redundancy in dMRI signals to estimate and remove noise‐dominant principal components from local patches. A breakthrough in the field occurred when Random Matrix Theory was used to derive data‐driven estimates for the threshold between noise and signal‐dominant principal components [12, 13]. This is achieved by fitting the Marchenko‐Pastur (MP) distribution [18] to the distribution of principal components, eliminating the need for a separate estimate of the noise variance [14, 19]. Refinements to this MPPCA approach for threshold estimation, which account for signal‐dominant components that alter the theoretical MP distribution, have been implemented to better estimate the signal‐noise boundary [10, 16, 20]. Additionally, to estimate noise‐free signal more accurately, optimal shrinkage of principal components is used to mitigate eigenvalue inflation due to noise [21] using nuclear [1] or Frobenius norms [5, 16]. Regardless of the exact implementation, it has been demonstrated that the domain (magnitude/complex) into which dMRI denoising is applied can have significant implications for subsequent analysis, affecting noise‐induced variance, bias, and true resolution in different ways [1, 2, 5, 22]. A challenge, however, is that across scanner vendors it is not straightforward (or not feasible at all in some cases) to control reconstruction parameters, such as apodization filters, or even access complex data, which would improve denoising outcomes.

In addition to these PCA‐based approaches, scanner manufacturers have recently introduced AI‐driven reconstruction and denoising solutions to enhance resolution and SNR while maintaining or reducing scan time [23, 24, 25, 26, 27]. These methods are integrated into the scanner reconstruction process, making them easily implementable in clinical protocols, and benefit from having access to unfiltered, complex domain data for optimal noise reduction. One such method is AIR Recon DL (ARDL) from GE HealthCare (Milwaukee, USA), a reconstruction and denoising convolutional neural network trained on millions of MRI images from various scan locations and acquisition methods, which is used to remove thermal noise and Gibbs ringing artifacts [23]. ARDL operates on 2‐dimensional image slices in the complex domain. The trade‐off between resolution, noise, and scan time typically governs acquisition choices for MRI, particularly for noise‐prone modalities such as dMRI, making it an ideal modality for assessing the performance of this denoising method. However, it is unclear how DL‐based approaches, such as ARDL, perform when compared against PCA‐based approaches.

In this paper, we address the above questions. Firstly, we develop an end‐to‐end offline reconstruction pipeline compatible with GE dMRI acquisitions. It takes raw, individual coil element complex k‐space data as input and provides access to complex dMRI data. The pipeline supports retrospective switching of filters and partial Fourier reconstruction methods and is compatible with in‐plane and out‐of‐plane accelerated acquisitions. This is necessary as scanner reconstruction on GE scanners does not provide access to complex dMRI data. Secondly, we demonstrate the application of this offline reconstruction pipeline by comparing PCA‐based denoising in the complex domain with GE's proprietary ARDL on standard PGSE acquisition from a wide‐bore scanner.

Specifically, we begin by validating the pipeline by comparing the scanner's and the pipeline's magnitude reconstruction across different acceleration and reconstruction parameters. We then compare ARDL with two commonly used denoising methods, NORDIC [11] and MPPCA_SVS_ [5, 12, 13] (MPPCA with singular value shrinkage), as they can be easily implemented in the complex domain. We also include MPPCA_SVS_ denoising in the magnitude domain to highlight improvements in denoising outcomes when using the offline reconstruction pipeline's complex‐domain data, over magnitude‐constrained denoising available from native scanner reconstruction. To directly evaluate denoising outcomes, we consider metrics of noise variance, noise floor bias, and proxies of noise distribution within the brain, as previously performed in [2]. As a secondary indirect comparison, we also evaluate the downstream effects of denoising through fiber orientation estimation and white matter bundle segmentation with probabilistic tractography.

## 2 Methods

### 2.1 Image Reconstruction Pipeline

We developed a comprehensive and scalable offline reconstruction pipeline for dMRI acquisitions on GE HealthCare scanners, starting from individual coil element k‐space data and producing coil‐combined images in both complex and magnitude domains. The pipeline can modify several reconstruction steps and handle multiple accelerated acquisition options.

**FIGURE 1:**
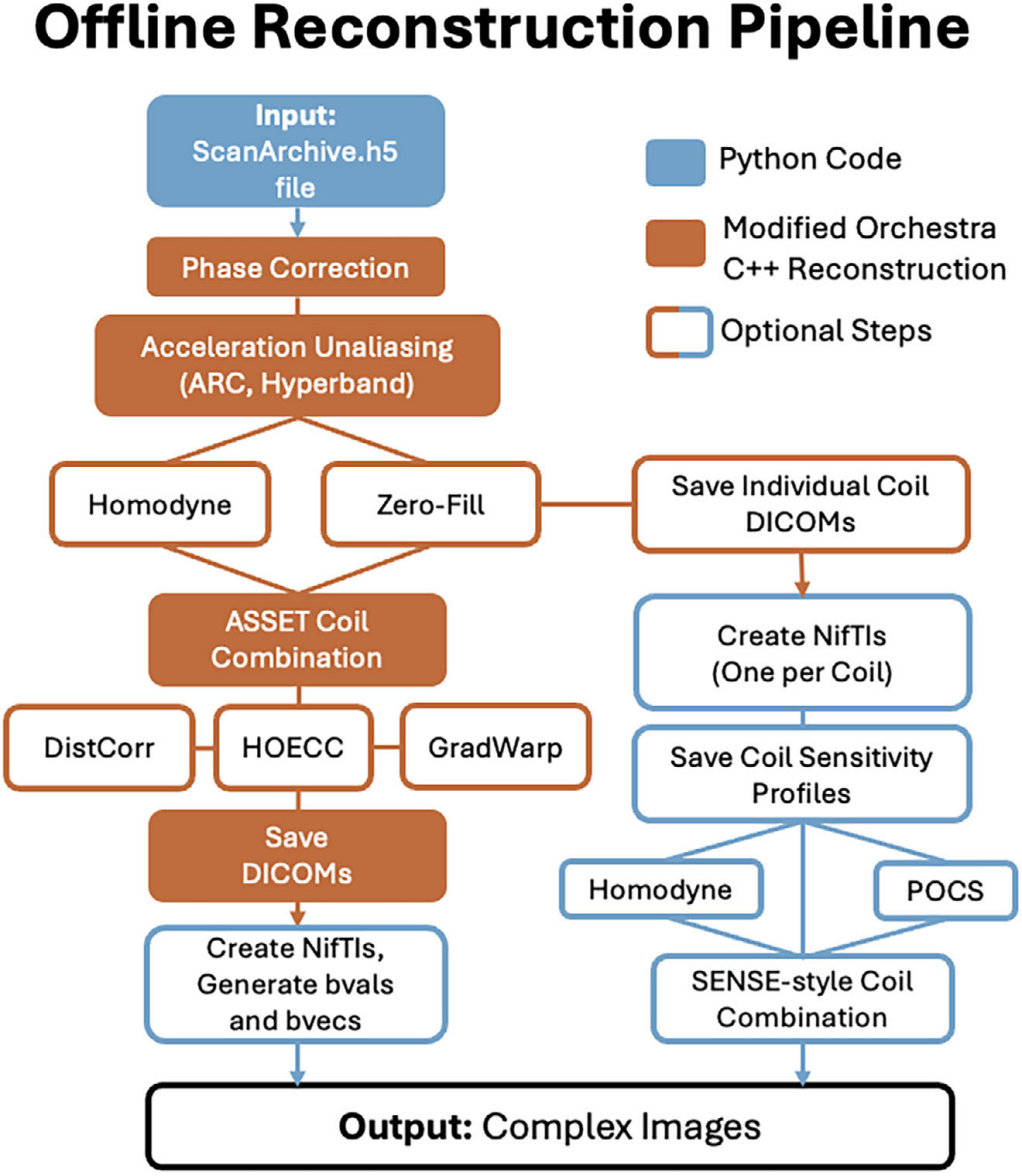
Outline of the offline reconstruction pipeline. Orange steps are modified C++ Orchestra SDK code; in blue are additional steps using custom Python code. Boxes with only colored borders (filled in white) are optional steps. The pipeline is implemented within a container for reproducibility and ease of use across operating systems.

We used Python (version 3.12.6) and built upon the C++ Orchestra software development kit (SDK), which provides simple example reconstruction scripts and is available through GE HealthCare's WeConnect website (https://weconnect.gehealthcare.com/) with the appropriate research license. The pipeline takes as input ScanArchive.h5 files (k‐space, individual coil data) and outputs DICOMs and NIfTI images (either magnitude or complex) as shown in Figure 1. Complementing further the capabilities of the default Orchestra SDK scripts, our workflow allows:

- Reconstruct coil‐combined complex (magnitude/phase or real‐rotated) data.
- Support of in‐plane (ASSET‐SENSE and ARC‐GRAPPA) and out‐of‐plane accelerations, with proper reconstruction of HyperBand acquisitions for simultaneous multi‐slice (SMS) accelerations.
- Retrospective reconstruction of partial Fourier acquisitions, using zero‐filling, POCS [28] and Homodyne [29] methods.
- Access to individual coil element complex images and extraction of coil sensitivity profiles to be used for sensitivity‐weighted coil combination.
- Proper indexing of dMRI volumes and slices for all combinations of in‐plane (SENSE‐like, GRAPPA‐like) and out‐of‐plane (SMS) accelerations.
- Optional flags to modify several reconstruction steps retrospectively, including switching on/off gradient non‐linearity correction, on/off apodization filters, on/off high‐order eddy current correction, and switching between complex (zero‐filling) and homodyne reconstruction [29] for partial Fourier acquisitions.
- Accounting for and preserving phase encoding direction and correct creation of diffusion b values (bval) and diffusion‐weighted direction vector (bvec) files for subsequent spin‐echo fieldmap‐based corrections of susceptibility‐induced distortions.

To enable the reproducible and straightforward implementation of the pipeline across various operating systems and high‐performance clusters, the offline reconstruction is embedded into Docker (https://docs.docker.com/) or Singularity/Apptainer (https://apptainer.org/) containers. Additionally, the C++ source code for the pipeline is available for customisation.

To evaluate and validate the capabilities of the pipeline, data using SENSE‐like acceleration (ASSET factors 1 and 2), GRAPPA‐like acceleration [30] (ARC factors 1 and 2), simultaneous multi‐slice [31, 32, 33] (HyperBand factors 1 and 4), and partial Fourier reconstruction methods (homodyne or zero‐filled) were acquired and reconstructed offline, using the developed reconstruction pipeline, as well as on the scanner. The magnitude reconstruction from both approaches was compared with validate the offline pipeline's output.

### 2.2 Denoising Comparisons

The offline reconstruction was developed with denoising in mind and hence offers the ability to perform denoising either in magnitude or complex domain at various stages of the reconstruction, for instance, prior to or after coil combination. In this work, we considered two PCA‐based denoising methods applied after coil combination:

- **MPPCA**_**SVS**_: This applies MPPCA with singular value shrinkage [21]. We used the default DESIGNER pipeline (v2.0.11) [34, 35] using 7 × 7 × 7 patches (using the smallest, odd N such that N × N × N > number of volumes acquired, as in [36]), which performs eigenvalue threshold estimation as in [10] with singular value shrinkage using Frobenius norm [21]. This was applied to both complex (real rotated homodyne [29]) data (MPPCA*_SVS_) and magnitude (|MPPCA|_SVS_) data.
- **NORDIC**: This is a variant of patch‐based denoising that spatially normalizes the data variance using a g‐factor map to ensure identical signal distribution across voxels [12]. We used the default 13 × 13 × 13 patches (so that the number of voxels in a patch and the number of diffusion volumes are approximately in a ratio of 11:1, as applied by the default NORDIC code) (https://github.com/SteenMoeller/NORDIC_Raw) [11] applied to complex (real rotated homodyne) data (NORDIC*). We did not provide noise‐only volumes; therefore, the default NORDIC method normalizes the data using an estimated g‐factor map using 2D MPPCA [12, 13] noise residuals, then calculates low‐rank thresholds using generated patch‐seized noise matrices.

We note that the same phase correction (real rotation using homodyne) is applied in the same way prior to complex denoising with either PCA method and that other variables such as patch size and patch averaging are varied across methods to match widely used implementation by developers of the methods.

**FIGURE 2:**
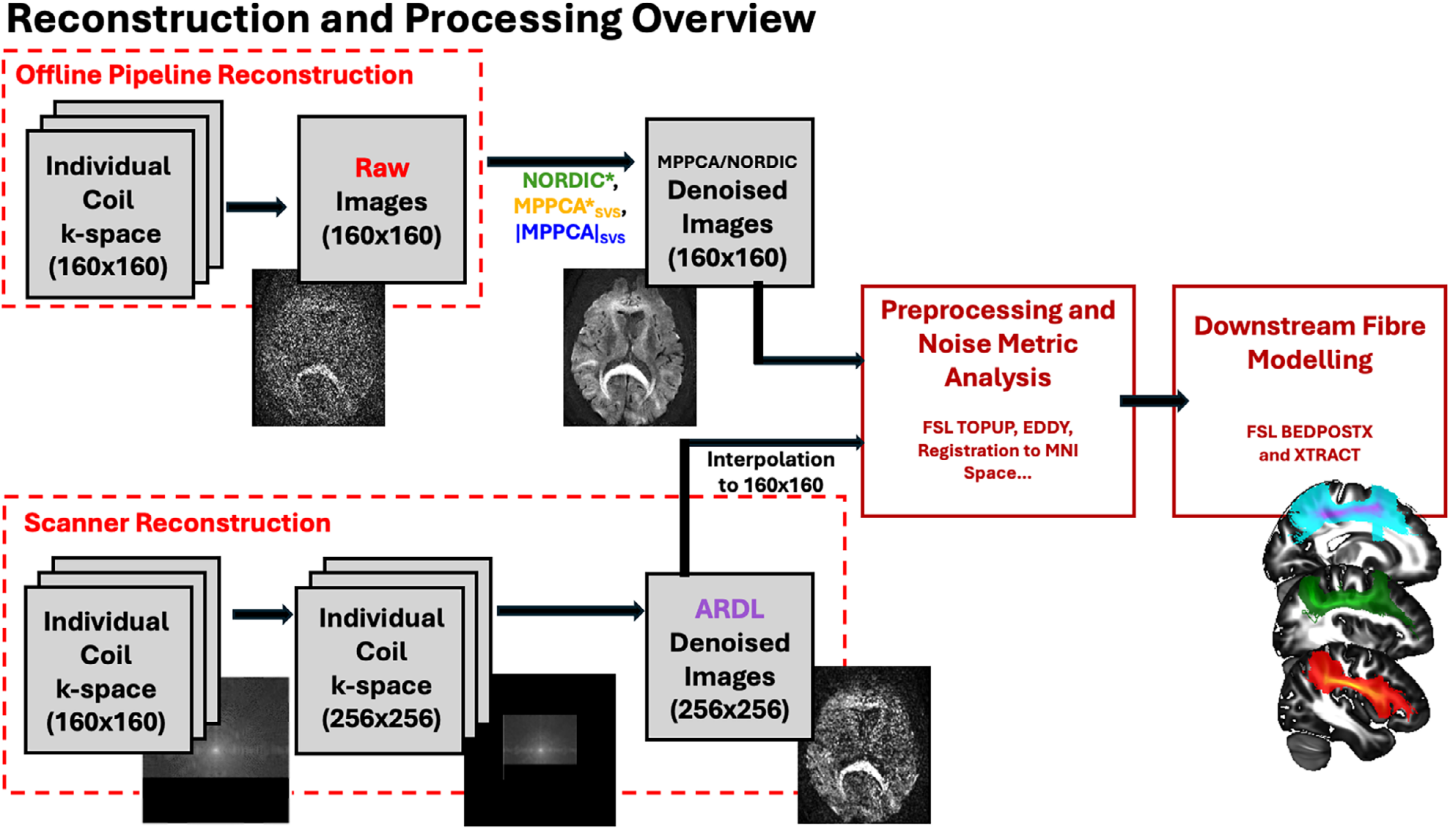
Overview of processing steps for comparison across denoising methods. For ARDL reconstruction and denoising, the k‐space is resampled to a 256 × 256 matrix and later (post‐ARDL) downsampled to the native matrix size.

We subsequently used the reconstruction pipeline to compare these PCA‐based denoising approaches with GE's AI‐based ARDL. Starting from raw k‐space data, we retained the real‐channel rotated data from homodyne reconstruction [29] (referred to as complex data in the rest of the paper) and used this for denoising in the complex domain. ARDL, which also operates in the complex domain, was applied with the “high” denoising weighting hyperparameter (from low, medium, and high options from the scanner console interface) on the same data, using retrospective reconstruction on the scanner. As ARDL denoising requires a fixed‐size 256 × 256 input matrix by design, k‐space was manipulated by placing the acquired k‐space in a zero‐filled 256 × 256 matrix during reconstruction. After ARDL was applied, data were downsampled to the acquisition matrix size using spline interpolation [37, 38]. An overview of the reconstruction and processing pipeline is shown in Figure 2.

### 2.3 Data Acquisition

Data for offline reconstruction pipeline validation were acquired on a GE HealthCare 3 T Premier wide‐bore scanner (Software Version MR29.2; GE HealthCare, Milwaukee, USA) at the University of Nottingham. Data from a single participant were acquired using 2 mm isotropic, 6 directions, *b* = 1000 s/mm^2^ PGSE‐EPI at various acceleration parameters: no acceleration (TR/TE = 8.0 s/57.2 ms), ASSET = 2 (TR/TE = 8.0 s/57.3 ms), ARC = 2 HyperBand = 4 (TR/TE = 8.0 s/59.7 ms) as well as a high‐resolution 1.3 mm isotropic HCP‐style data [39, 40], (98 volumes/shell, two shells: *b* = 1500, 3000 s/mm^2^, HyperBand = 4, TR/TE = 4.8 s/88.3 ms). All data were acquired using a 48‐channel head coil.

Data for denoising comparisons were acquired at the Cambridge University Hospitals NHS Foundation Trust (Cambridge, UK) for four healthy participants (two female, age range 27 to 54) on a GE HealthCare 3 T Premier wide‐bore scanner with a 48‐channel head coil (Software Version MR30.1; GE HealthCare, Milwaukee, USA). Data were acquired using a PGSE‐EPI sequence following the acquisition protocols used in the Human Connectome Project (HCP) [39, 40], with 1.25 mm isotropic voxel size, 98 volumes/shell, two shells: *b* = 1500, 3000 s/mm^2^, and 14 *b* = 0 s/mm^2^ volumes throughout the acquisition with one reverse gradient (AP/PA) *b* = 0 s/mm^2^ acquired prior to the main data, HyperBand = 4, TR/TE = 4.8 s/88.3 ms, 60% partial Fourier with Homodyne reconstruction. A 1 mm isotropic T1‐weighted MPRAGE with higher order shimming was acquired to create white matter masks and improve registration to MNI templates.

All data were acquired after informed consent was obtained from all participants. Ethical approval was respectively obtained from the University of Nottingham Faculty of Medicine and Health Sciences Research Ethics Committee (B12012012a_073) and from the East of England regional review board, Cambridgeshire and Hertfordshire Research Ethics Committee (08‐H0311‐117).

After acquisition, raw data involving the 48 individual head coil channels were exported and reconstructed using the offline pipeline to coil‐combined images.

### 2.4 Pre‐Processing and Analysis

Post‐denoising, identical distortion correction across denoising methods was carried out using a standard FSL‐eddy pipeline (FSL v6.0.6.4) [41, 42] on the images, including susceptibility‐induced, eddy‐current distortion and motion correction. We evaluated several aspects of denoising impact, from raw data to downstream analysis, as recommended in Manzano‐Patron et al. [2]. To assess denoising efficacy with respect to variance suppression, SNR and angular CNR were obtained using FSL‐eddyQC [43]. To assess efficacy with respect to bias and noise floor suppression, we examined the distribution and dynamic range profile of the signal, before and after denoising. To account for noise spatial non‐stationarities, noise distributions were estimated from inside the brain (rather than background) using maximally attenuated (i.e., high *b*‐value) CSF signal from the ventricles [44]. Noise‐floor‐induced signal rectification was assessed by examining the signal attenuation of parallel vs. perpendicular (i.e., maximally vs. minimally attenuated) to principal fiber directions (DTI V_1_) in highly anisotropic voxels (FA > 0.6 across all methods) [44], in the corpus callosum, using single‐subject warped masks. To account for signal intensity changes caused by the interpolation of ARDL images, signal intensities of ARDL were renormalised, using the ratio of mean S0 signal between |RAW| and ARDL in the ventricles. Secondary, downstream effects of denoising methods were also assessed through fiber orientation estimation and probabilistic tractography, carried out using FSL's bedpostX [45, 46] and XTRACT [47] for each denoising mode and we compared session‐wise tract density maps with average probabilistic tract density maps from a 50‐subject HCP average [47].

## 3 Results

### 3.1 Offline Reconstruction Features and Validation

**FIGURE 3:**
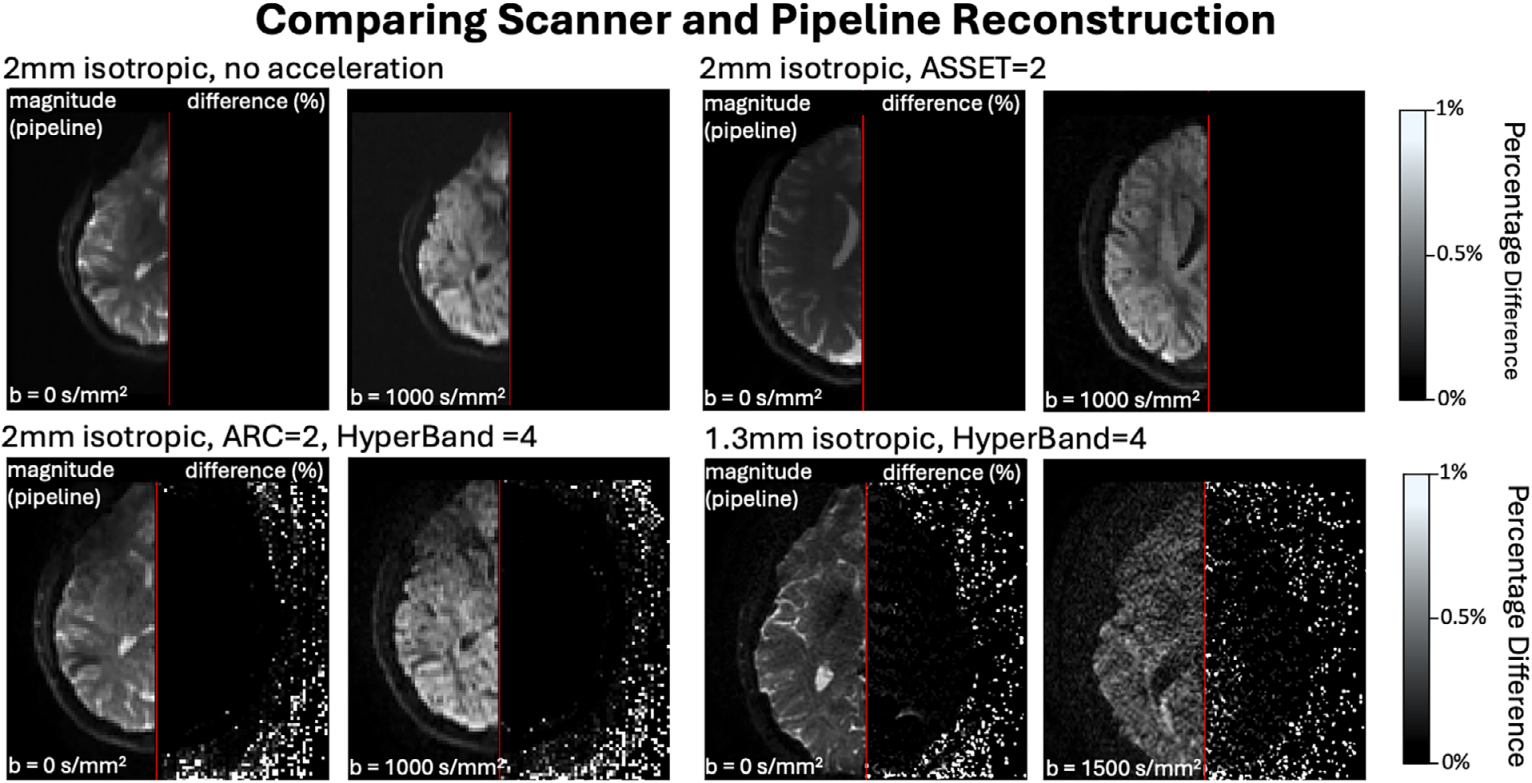
Comparison of offline magnitude and scanner reconstruction. Offline pipeline reconstruction and voxel‐wise percentage difference in signal intensity between offline and scanner reconstruction for (top left to bottom right) 2 mm isotropic no acceleration, ASSET = 2, ARC = 2 + HyperBand = 4 and 1.3 mm isotropic HyperBand = 4 data. Minimal differences are observed when no acceleration or ASSET is used, and differences are below 1% in absolute value when HyperBand or ARC is used.

We validated the offline reconstruction by comparing magnitude images between the scanner and the offline reconstruction pipeline, as complex dMRI data are not available from the scanner reconstruction. We performed comparisons across resolutions and b‐values, as shown in Figure 3, for various in‐plane (no acceleration, ASSET = 2, and ARC = 2) and out‐of‐plane (no HyperBand and HyperBand = 4) acceleration. In all cases, the offline magnitude reconstruction matched the scanner reconstruction almost perfectly when no SMS acceleration was used. When SMS acceleration was used, minor signal differences were seen within the brain, but these differences were below 1% in absolute value. Further validation of the offline reconstruction pipeline is provided in supplementary Figure S1, using data acquired at a different site with a different software version (MR30.1), across four additional sessions.

**FIGURE 4:**
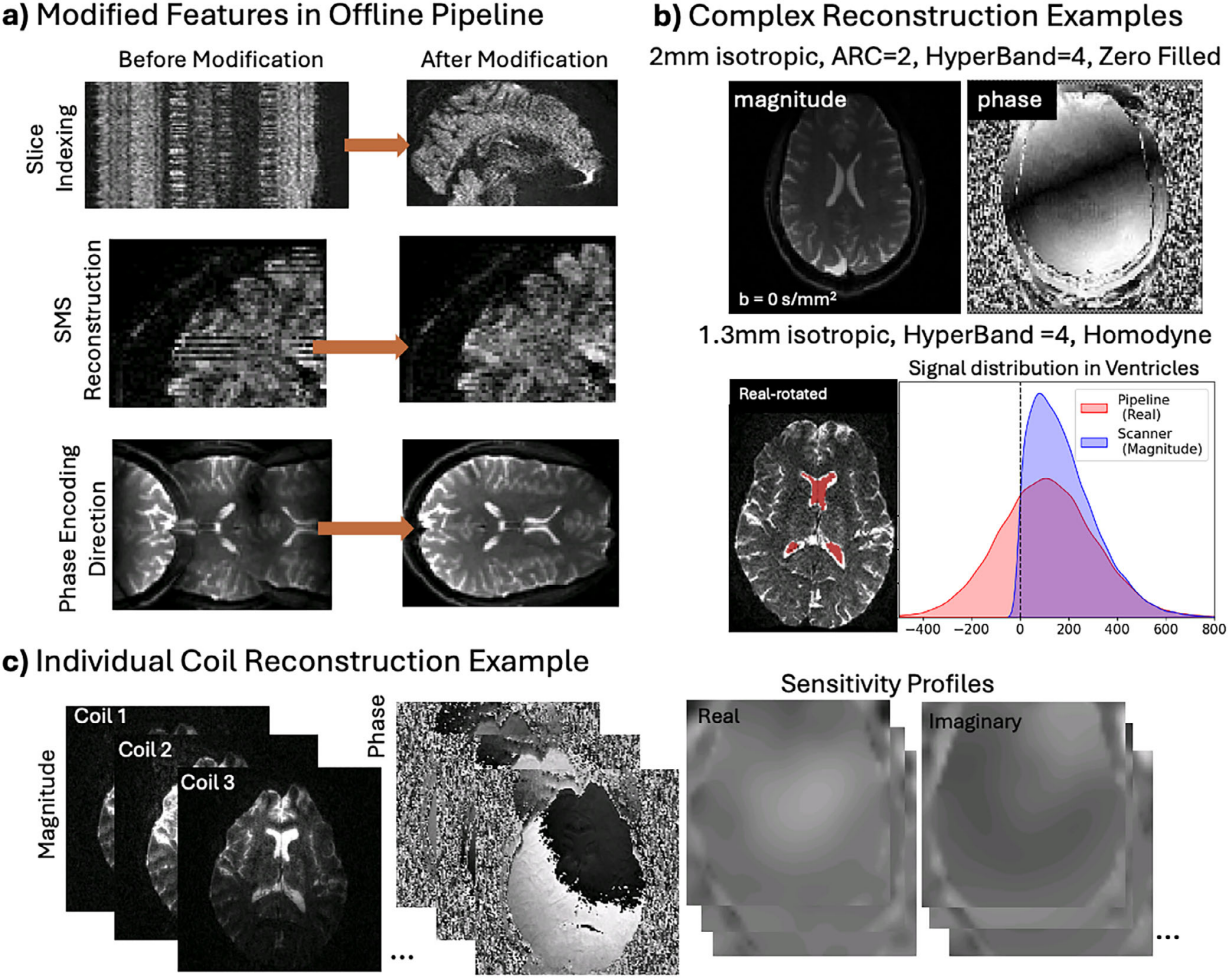
Offline reconstruction modifications and additional features. (a) Images highlighting some modifications to the original Orchestra SDK reconstruction, from top to bottom: Correcting slice indexing, SMS reconstruction and phase encoding direction reconstruction. (b) Examples of pipeline complex zero‐filled (magnitude and phase) or homodyne (real rotated) reconstruction, not available from scanner reconstruction. (c) Example of individual coil reconstruction showing single coil magnitude, phase and sensitivity profiles for coil combination.

Figure 4a highlights some modifications made to the C++ Orchestra SDK to enable correct indexing of slices, correctly account for the reference scans in SMS acquisitions, and correctly identify the phase encoding direction for blip‐reversed acquisitions. We showcase examples of reconstructed complex images in Figure 4b, showing magnitude and phase reconstruction for a zero‐filled acquisition and real‐rotated homodyne acquisition (exemplified by negative values in the CSF signal). The offline pipeline also gives access to complex individual‐coil images and respective coil sensitivity profiles, as shown in Figure 4c, which can be retrospectively reconstructed using custom homodyne or POCS algorithms.

### 3.2 Denoising Comparisons

**FIGURE 5:**
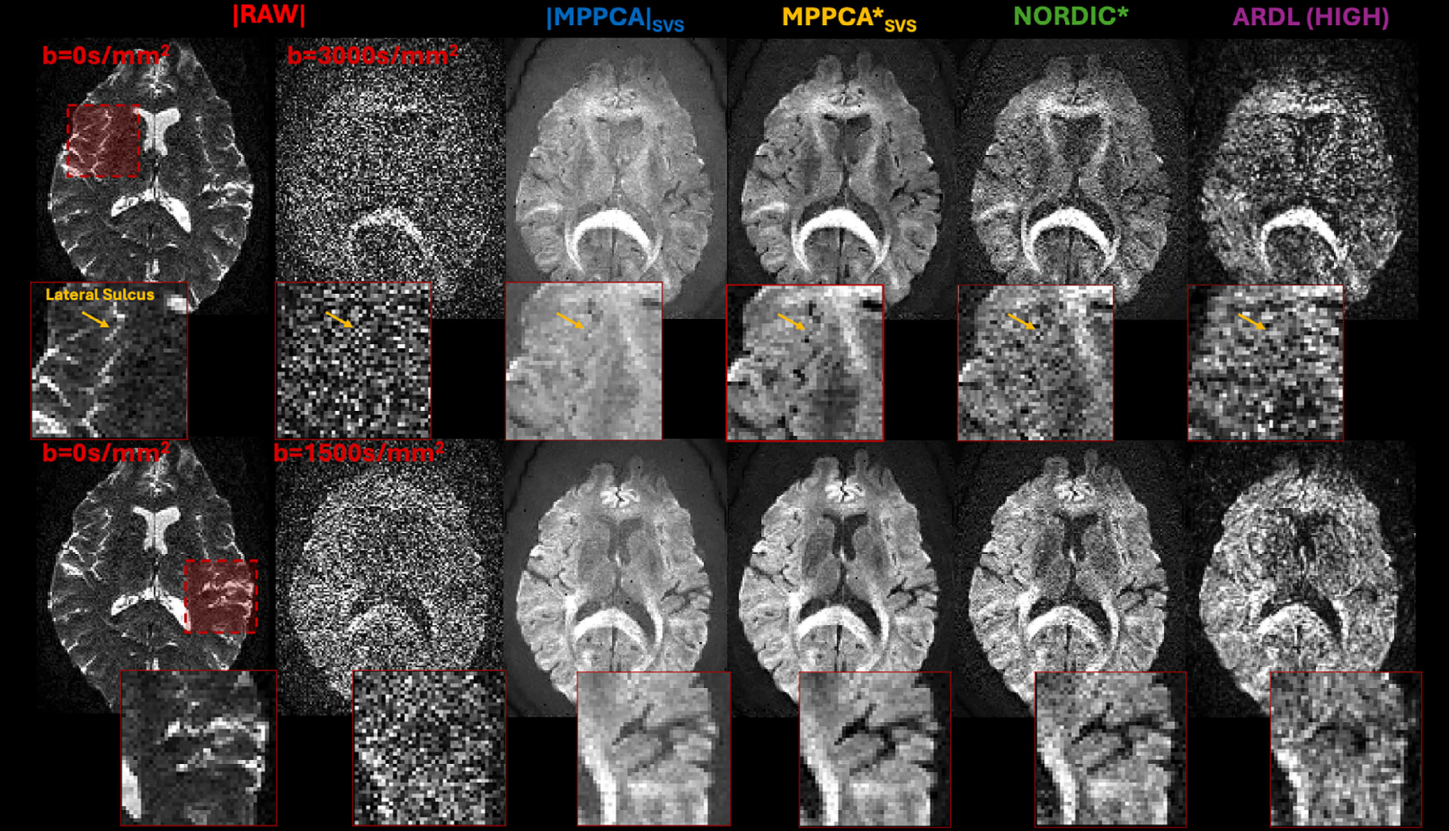
Comparison of raw and denoised data for *b* = 3000 s/mm^2^ (top) and *b* = 1500 s/mm^2^ (bottom) slices. A raw *b* = 0 s/mm^2^ slice is shown on the left to provide anatomical benchmarks for diffusion weighted volumes. Different columns correspond to raw data, magnitude‐denoised |MPPCA|_SVS_, complex‐denoised MPPCA*_SVS_, complex‐denoised NORDIC* and complex‐denoised ARDL with high denoising weighting. Smaller panels highlight sulci (highly visible in *b* = 0 s/mm^2^ raw data) to compare improvements in resolution and anatomical fidelity of denoised data.

The effect of denoising on exemplary slices for *b* = 3000 s/mm^2^ (top) and *b* = 1500 s/mm^2^ (bottom) shells is shown in Figure 5. To showcase the increased ability to discriminate anatomy post‐denoising, sulci between the insula and adjacent lobes are highlighted. The location and size of the sulci is compared between high signal CSF in *b* = 0 s/mm^2^ slices and low signal in diffusion weighted slices. All denoising approaches substantially improve the images over the raw data. MPPCA_SVS_ denoised diffusion slices show the highest anatomical correspondence with the *b* = 0 s/mm^2^ slice. Noise floor‐induced bias in magnitude denoised data (|MPPCA|_SVS_) is highly visible when compared with complex denoised data (MPPCA*_SVS_, NORDIC or ARDL), highlighting the advantages of denoising complex, pipeline‐reconstructed data over magnitude‐denoised data. Furthermore, as highlighted in the figure inlays, anatomical details are more visible when applying MPPCA*_SVS_ or NORDIC compared with ARDL, with MPPCA*_SVS_ showing qualitatively the best performance across all approaches.

**FIGURE 6:**
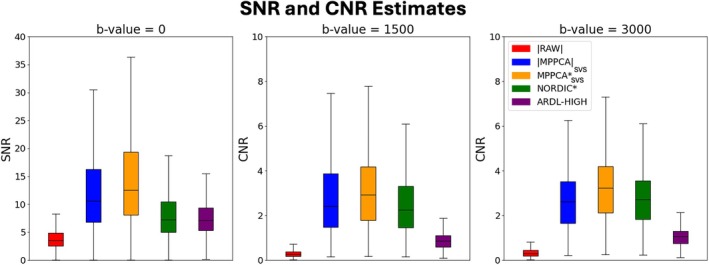
SNR (across *b* = 0 s/mm^2^ volumes) and angular CNR estimates using FSL‐EddyQC.

**FIGURE 7:**
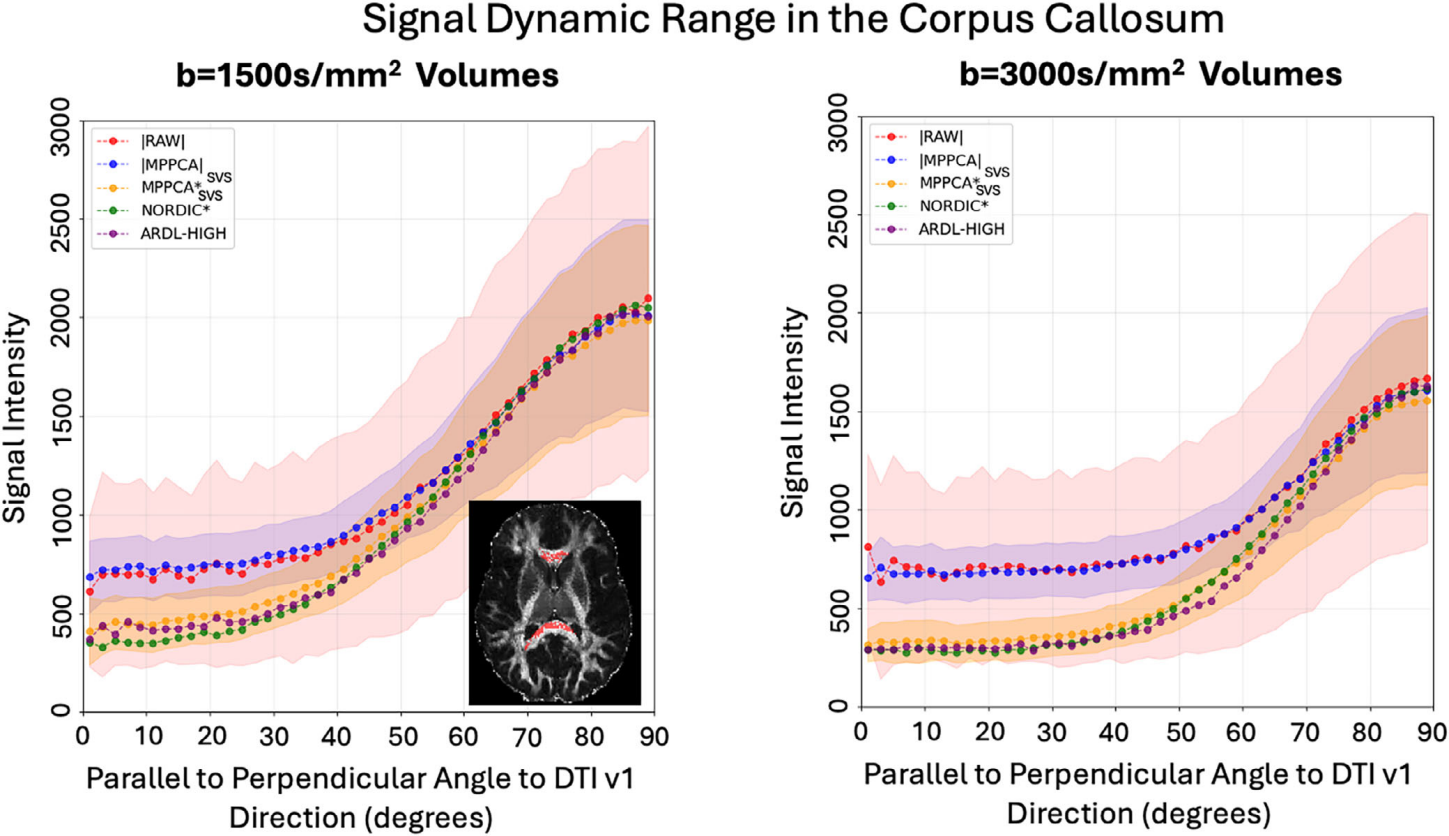
Single‐subject signal dynamic range in the corpus callosum obtained using a registered Corpus Callosum mask and an FA > 0.6 mask from all methods (panel highlights example high FA voxel mask in red). Left—Signal attenuation measured by binning *b* = 1500 s/mm^2^ shell signals with respect to angle to DTI v1 direction. Right—Signal attenuation measured by binning *b* = 3000 s/mm^2^ shell. ±SD ranges are shown at each bin angle for |RAW|, |MPPCA|, and MPPCA* data, highlighting the reduction in variance when denoising is applied.

**FIGURE 8:**
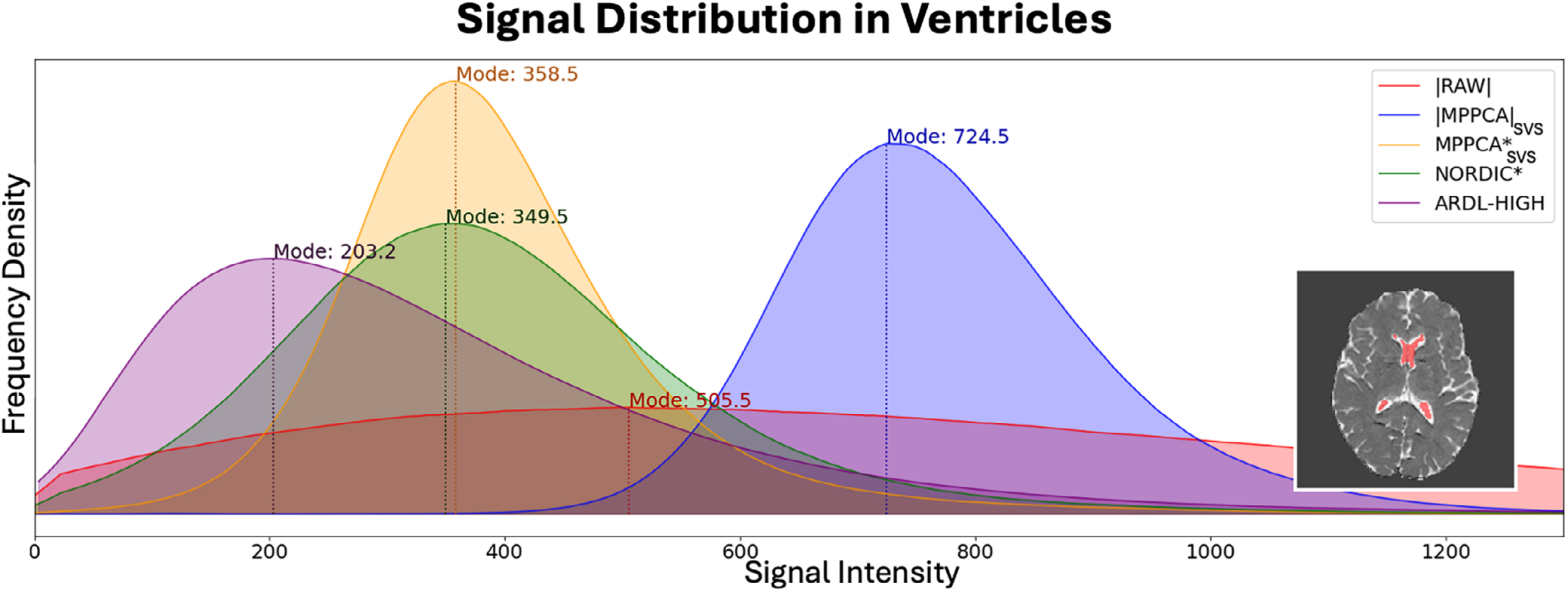
Distribution of signal in the ventricles as a proxy for the noise distribution. Inlay highlights example ventricle mask (red) for a single subject.

We subsequently performed quantitative assessments. Figures 6, 7, 8 provide an overview of metrics related to changes in variance and bias of the signal due to denoising. Estimates of SNR across the *b* = 0 *s*/mm^2^ volumes and angular CNR per shell are presented in Figure 6, showing better performance for 4D PCA‐based denoising methods compared with 2D ARDL. Single‐subject SNR and CNR boxplots are shown in Figure S2.

Figure 7 presents the dMRI signal attenuation at a given *b* value, having the signal reordered based on the dot product of the corresponding diffusion‐sensitisation gradient direction and the principal DTI V_1_ in each voxel (i.e., parallel to perpendicular the main fiber orientation) and then averaged across a region of very anisotropic voxels (depicted by the mask in the body of the corpus callosum). We can observe how the signal is rectified in the magnitude domain and how all complex‐domain denoising methods significantly increase the signal dynamic range and reduce the noise floor bias. Furthermore, standard deviation ranges are shown for |RAW|, |MPPCA_SVS_|, and MPPCA*_SVS_ data for each one‐degree angle bin, showing the reduction in variance of signal after denoising. Patterns from a single subject are depicted in Figure 7, but similar trends are shown for all subjects in Figure S3.

Finally, Figure 8 presents the distribution of maximally attenuated CSF signal in the ventricles, a proxy for the noise distribution, again showing increased noise floor suppression when denoising in the complex domain, with ARDL having the best performance. Taken together, these results suggest that denoising in the complex domain reduces noise‐induced bias and variance, with ARDL performing the best in the removal of bias in the ventricles, and MPPCA*_SVS_ performing the best in the reduction of variance.

**FIGURE 9:**
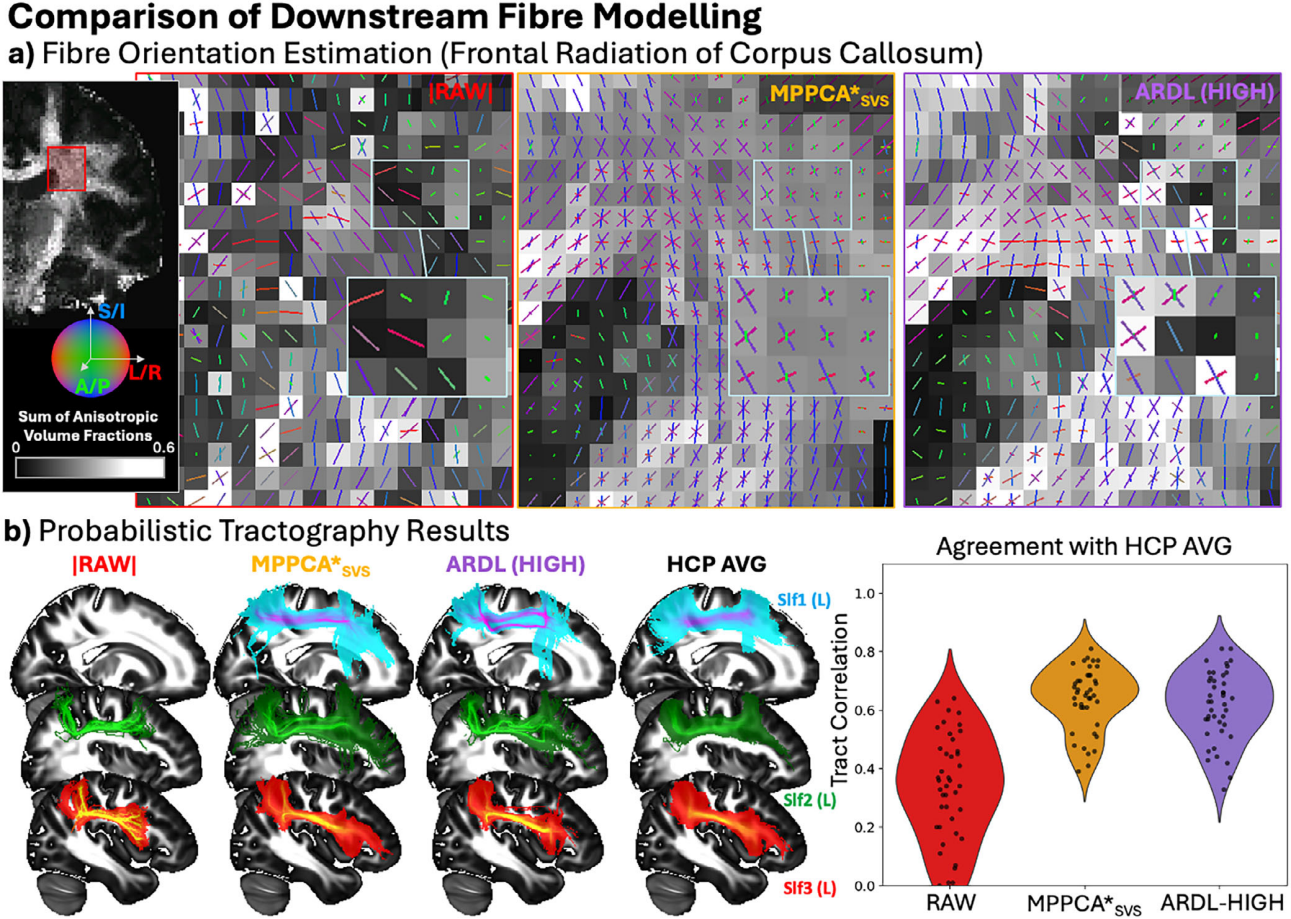
(a) Comparison of fiber orientation estimation, using FSL's BedpostX, highlighting regions of complex fiber architecture involving multiple crossing fibers. Sticks are length‐scaled by their corresponding volume fraction. Voxel shade represents the sum of anisotropic volume fractions. (b) Results from probabilistic tractography (average of 4 subjects) using FSL's XTRACT showing maximum intensity projections of superior longitudinal fasciculi (left) and correspondence with a 50‐subject HCP average for all 42 tracts (right). Path distributions were thresholded at 0.1% and correlated against the corresponding path distributions of the HCP average.

Secondary, downstream effects of denoising were assessed by exploring fiber orientation estimation and white matter bundle reconstructions, as seen in Figure 9. Fiber orientation estimations using the multi‐shell ball & sticks model (up to 3 orientations per voxel) are shown in Figure 9a, highlighting estimates around the centrum semiovale, as a location of complex (up to three‐way) fiber crossings. Both MPPCA*_SVS_ and ARDL denoised data show increased identification of two and three‐way crossings, depicting orientations from the corpus callosum, the corticospinal tract and the longitudinal fasciculi running in and out of the coronal plane. These are mostly absent in the raw data. Also, MPPCA*_SVS_ estimates are more qualitatively spatially continuous than ARDL, implying a better characterization of the underlying tissue. It is important to note that FSL's BedpostX uses an automatic relevance detection (ARD) algorithm [48] to select the number of crossing fibers in a voxel, using a data‐driven approach to select between one and three fibers to model each voxel's signal.

Fiber orientation estimates were subsequently used to perform probabilistic tractography and reconstruct 42 major white matter fiber bundles using FSL's XTRACT. Figure 9b (left panel) examples participant‐averaged tract density maps, highlighting results for the superior longitudinal fasciculi (SLF1, SLF2 and SLF3) and 50‐subject HCP averages for comparison. We selected the SLFs as they pass through regions of complex fiber architecture. In the RAW data, tracking of the SLF1 was unsuccessful, whereas MPPCA*_SVS_ and ARDL successfully tracked the SLF1, though ARDL incorrectly fit streamlines which more likely belong to SLF2. Other smaller differences can be observed, for instance MPPCA*_SVS_ reconstructed SLF3 projects more anteriorly than the RAW or ARDL reconstruction. To quantify the differences further, violin plots on the right show the Pearson's correlation between all 42 tract density maps of raw or denoised data and those of the HCP average, used as a reference. Denoising both with MPPCA*_SVS_ and ARDL significantly improved correspondence to the reference, with MPPCA*_SVS_ yielding better agreement overall.

## 4 Discussion

In this work, we presented a reconstruction pipeline for GE dMRI acquisition, which uses individual coil k‐space data to give access to unfiltered, coil‐combined complex data. The pipeline also gives the freedom to alter reconstruction steps like apodization filters, partial Fourier reconstruction method (POCS/Homodyne/Zero‐filled), and gradient warp fields. In line with previous findings which used data from other manufacturers [1, 2, 5, 22], we demonstrate the benefits of denoising in the complex domain, using data obtained with the proposed pipeline, over magnitude‐constrained denoising. This is particularly evident in reducing the effect of the noise floor bias, as observed in the signal dynamic range in the corpus callosum, and noise distribution in the ventricles. Our offline reconstruction allowed us to obtain high‐quality data at high spatial resolution and *b* value (1.25 mm isotropic, *b* values up to 3000 s/mm^2^), similar to HCP dMRI [39], even if using a wide‐bore scanner, such as the GE Premier, and a standard PGSE EPI sequence.

The offline reconstruction pipeline is deployable in a containerized implementation, enabling reproducible outcomes across operating systems. However, its reconstruction speed is slower than on‐scanner reconstruction, particularly when implementing computationally intensive algorithms, such as simultaneous multi‐slice accelerations. For an individual experimental dataset used in this study of 1.25 mm isotropic resolution and 198 volumes, on‐scanner reconstruction took approximately 12 min and could be completed during acquisition, whereas the offline pipeline required over 4 h. However, the on‐scanner reconstruction is an optimized product with GPU support, whereas our offline reconstruction is a CPU‐based prototype that can be further optimized. In addition, GE HealthCare scanners support the automatic transfer of raw data files to a remote host, which enables remote processing at the end of an acquisition. This enables the offline pipeline to be integrated into a larger remote automated processing pipeline, where multiple reconstructions can run in parallel on external compute resources. As the offline pipeline does not rely on scanner hardware, this approach is scalable to larger clinical cohorts despite the longer per‐dataset reconstruction time.

Previous work reports that denoising dMRI data reduces the variability of downstream metrics across scanners and protocols, with the greatest benefit when denoising in the complex domain [5]. So far, these comparisons have been limited to a single manufacturer across denoising domains [5], or using multiple manufacturers but constrained to magnitude domain denoising, with little control of the reconstruction algorithms used across vendors [49]. Our introduction of a flexible offline reconstruction pipeline for GE dMRI data allows us to bridge the gap and allow cross‐vendor and cross‐domain comparisons in future work.

We compared the performance of proprietary ARDL denoising and reconstruction, which benefits from within scanner integration and is modality agnostic, to the offline reconstruction pipeline alongside commonly used PCA‐based denoising methods [10, 11, 13]. These results suggest that PCA‐based denoising methods outperform ARDL in terms of identifying fine image details and contrast‐to‐noise ratio gains. Indirect denoising evaluation using fiber orientation estimation shows that PCA‐based methods yield results that are more consistent with known anatomical fiber architectures and show better agreement with high‐resolution HCP tractography averages than ARDL. This is not surprising, given that ARDL is 2D. However, ARDL also operates in the complex domain, and the overall benefits in reducing noise‐induced biases agree with the benefits offered by PCA‐based denoising approaches when applied to complex data.

ARDL requires a 256 × 256 input matrix size which was achieved by embedding the acquisition matrix (160 × 160) into a zero‐filled oversized matrix, followed by a post‐ARDL spline interpolation to the acquisition matrix size for comparison against the PCA‐based denoising methods. To ensure our trends were not driven by our custom post‐ARDL interpolation, we carried out a comparison where all denoising methods were zero‐filled to a 256 × 256 matrix size, using the same processing as the ARDL preprocessing, followed by the spline interpolation to the acquisition matrix size (160 × 160) prior to denoising. The trends in our results remained unchanged, as shown in Figure S4. Additionally, the comparisons carried out in this paper only highlight the performance of ARDL on the ‘high’ setting; Figure S5 shows that this was the best performing mode available from scanner selection (low, medium, high). However, a limitation of this work is that we did not test ARDL capabilities beyond the ‘high’ setting, as ARDL uses a tuning hyperparameter ranging from 0 to 1, and the high setting corresponds to 0.75. Additionally, ARDL includes unringing features, which were not evaluated against alternatives with publicly available source code [50] in this study.

Previous work has suggested that denoising earlier in the reconstruction process is beneficial to denoising outcomes [1, 51], from the reduction of non‐stationary g‐factor contributions to noise [11, 52]. Although ARDL benefits from denoising individual coil complex data rather than coil‐combined data, it is important to note that 2D ARDL does not utilize the full redundancy of 4D dMRI data, unlike the PCA‐based approaches, developed explicitly for use in dMRI data. The proposed pipeline provides access to individual coil complex data, enabling evaluations of denoising earlier in the reconstruction process and across partial Fourier methods. Additionally, previous work has shown that by using additional redundancy from the use of all individual coils can reduce further the effect of noise bias [1, 4], this was not tested in this work but can be readily implemented within the proposed pipeline.

We showed that complex MPPCA_SVS_ performs better than NORDIC on these GE data in terms of contrast‐to‐noise gain and in the ability to resolve fine anatomical details post‐denoising. This is seemingly different to previous results when comparing these two approaches with Siemens datasets [2], with the same implementations of patch sizes. The discrepancies in observed trends are likely linked to the differences in MPPCA denoising methods used, as shown in Figure S6. Specifically, the MPPCA implementation in previous work (MRtrix v3.0.7) [36] did not include singular value shrinkage correction to compensate for noise‐induced inflation of eigenvalues [21], patch averaging, or the inclusion of symmetric thresholds for identifying signal eigenvalues. In contrast, these features are included in the DESIGNER implementation [34, 35] used in the present study. In the presence of noise, eigenvalues are inflated, as described in previous work [21]. Applying singular value shrinkage improves the estimation of noise‐free principal components from a noisy distribution. Furthermore, the use of eigenvalue thresholds based on symmetric evaluation of the singular values more accurately estimates the cutoff between noise and signal eigenvalues in empirical data as examined by Cordero Grande et al. [10] and Olesen et al. [16]. By comparison, NORDIC does not use singular value shrinkage and calculates eigenvalue thresholds from Monte Carlo simulations of noise. In the absence of a sample noise acquisition, NORDIC employs a 2D MPPCA approach (without singular value shrinkage or symmetric eigenvalue thresholds) with large patch sizes (15 × 15) to estimate noise. The estimate is then used to normalize image intensities prior to Monte Carlo noise simulations. Consequently, when no sample noise acquisition is available, NORDIC is restricted to a more basic MPPCA‐based noise estimation strategy. Taken together, these methodological differences likely explain the superior performance of MPPCA_SVS_ observed here relative to both NORDIC and MPPCA_MRtrix_ and highlight the importance of singular value shrinkage and symmetric eigenvalue thresholding in effective PCA‐based denoising.

Additionally, the previous work used Siemens data as opposed to GE data, and there are well known cross‐vendor differences in partial Fourier reconstruction that could also explain some differences in denoising outcomes [51]. Siemens reconstruction often uses zero‐filled or POCS methods, whereas GE most commonly uses Homodyne reconstruction with a lower partial Fourier fraction. The downstream denoising consequences of vendor‐specific partial Fourier reconstruction methods and their fractions are not well explored. However, even within a single vendor, these choices have been shown to significantly affect denoising outcomes [51].

A limitation in our work is that we could not consider or understand effects and limitations of ARDL with respect to the network architecture implemented or training data used. This information is not published in the literature or known to the authors. Therefore, our comparisons aimed to evaluate ARDL, which is approved for clinical use and available commercially across all GE systems, against denoising methods commonly used in the scientific literature and dMRI processing pipelines. Furthermore, our comparisons only involved healthy subjects, not considering potential limitations of ARDL subject to restrictive training data. Nevertheless, it is important to note that ARDL has US FDA approval and is CE marked for use across all anatomies and contrasts, and is scale invariant, suggesting that the model is trained on statistical features of the data and not anatomically specific structures. Therefore, results found in this paper should be generalizable to clinical cohorts or depict a best‐case scenario for ARDL as a minimum. In other words, if ARDL was to perform worse in clinical cohorts than the healthy participants used in this study, the trends reported here (that complex‐domain patch‐based denoising methods perform better than ARDL) would still be valid.

The primary aim in developing the offline pipeline was to reproduce vendor‐specific reconstruction outside the scanner environment, enabling validation against scanner outputs while providing increased flexibility for research use, as demonstrated by the performed comparisons in this study. This approach allows the use of reconstruction methods perceived by manufacturers to extract what is optimal performance from their hardware. At the same time, it is important to support alternative reconstruction and preprocessing strategies that are publicly available, transparent, and potentially harmonizable across vendors. To this end, the pipeline also provides access to individual coil data, enabling the use of open, vendor‐agnostic reconstruction and denoising methods [53, 54]. For future work on other MRI platforms, we anticipate that offline reconstruction pipelines will benefit from incorporating both vendor‐specific reconstructions for validation and comparison, as well as open, cross‐vendor methods to facilitate harmonization and methodological transparency.

## 5 Conclusion

Our offline reconstruction pipeline for GE dMRI acquisition was validated against scanner magnitude reconstruction and gives access to unfiltered complex domain data for acquisitions with in‐plane and SMS acceleration. We used it to demonstrate potential for significant gains in dMRI data quality, allowing denoising in the complex domain, both for reducing noise‐induced variance and bias. PCA‐based (4D) methods outperformed proprietary 2D complex‐domain denoising using deep learning (ARDL) in terms of qualitative resolution, reduction of noise‐floor bias, and variance.

## Funding

This work was supported by GE Healthcare, The University of Nottingham, and the European Research Council, 101000969.

## Conflicts of Interest

GE HealthCare partially funds Francesco D'Antonio's PhD studentship.

## Supporting information


**Figure S1:** Validation of the on‐scanner and offline pipeline reconstruction using experimental data from four subjects acquired for denoising comparison (1.25 mm isotropic, Hyperband factor 4). Results are consistent with those shown in Figure 3, demonstrating high correspondence between reconstructions within the brain, with noisy differences mostly present outside the brain. Importantly, these results originate from a different site with a different software version (MR30.1) than those presented in Figure 3.
**Figure S2:** Single‐subject SNR (across *b* = 0 s/mm^2^ volumes) and angular CNR estimates using FSL‐EddyQC.
**Figure S3:** Single‐subject signal dynamic range in the Corpus Callosum (*b* = 1500 s/mm^2^—top, *b* = 3000 s/mm^2^—bottom).
**Figure S4:** Comparison of denoising outcomes with identical interpolation steps across methods using a single subject. Denoising using patch‐based approaches is carried out after the 256 × 256 to 160 × 160 step. (a) SNR and CNR evaluation. (b) Signal dynamic range in the corpus callosum normalized with respect to highest angle bin signal value. (c) Comparison of resolution outcomes.
**Figure S5:** Comparison of denoising outcomes across ARDL settings (low—0.3, medium—0.5, high—0.75) using a single subject. (a) SNR and CNR evaluation. (b) Signal dynamic range in the corpus callosum normalized with respect to highest angle bin signal value. (c) Comparison of resolution outcomes.
**Figure S6:** Qualitative comparison of |RAW|, MPPCA*_SVS_, MPPCA*_MRtrix_, and NORDIC* on a *b* = 3000 s/mm^2^ slice.

## Data Availability

The offline reconstruction pipeline is available through GE HealthCare's WeConnect website (https://weconnect.gehealthcare.com/) with the appropriate research license. The data that support the findings of this study are available from the corresponding author upon reasonable request.
