# Supplementary material for: Offline Reconstruction of Diffusion MRI Acquisitions for Comparison Between Complex PCA‐Based and AI‐Based Denoising
Source: Magn Reson Med. 2026 Mar 7;96(1):435–47. doi: 10.1002/mrm.70336 (PMC13156438; doi:10.1002/mrm.70336)
Supplement: Supplementary file 1 — Figure S1: Validation of the on‐scanner and offline pipeline reconstruction using experimental data from four subjects acquired for denoising comparison (1.25 mm isotropic, Hyperband factor 4). Results are consistent with those shown in Figure 3, demonstrating high correspondence between reconstructions within the brain, with noisy differences mostly present outside the brain. Importantly, these results originate from a different site with a different software version (MR30.1) than those presented in Figure 3. Figure S2: Single‐subject SNR (across b = 0 s/mm2 volumes) and angular CNR estimates using FSL‐EddyQC. Figure S3: Single‐subject signal dynamic range in the Corpus Callosum (b = 1500 s/mm2—top, b = 3000 s/mm2—bottom). Figure S4: Comparison of denoising outcomes with identical interpolation steps across methods using a single subject. Denoising using patch‐based approaches is carried out after the 256 × 256 to 160 × 160 step. (a) SNR and CNR evaluation. (b) Signal dynamic range in the corpus callosum normalized with respect to highest angle bin signal value. (c) Comparison of resolution outcomes. Figure S5: Comparison of denoising outcomes across ARDL settings (low—0.3, medium—0.5, high—0.75) using a single subject. (a) SNR and CNR evaluation. (b) Signal dynamic range in the corpus callosum normalized with respect to highest angle bin signal value. (c) Comparison of resolution outcomes. Figure S6: Qualitative comparison of |RAW|, MPPCA*SVS, MPPCA*MRtrix, and NORDIC* on a b = 3000 s/mm2 slice. [file MRM-96-435-s001.docx]

**Supplementary Material**

**Offline Reconstruction of Diffusion MRI Acquisitions for Comparison Between Complex PCA-based and AI-based Denoising**

Francesco D’Antonio, Shaun Warrington, Jose-Pedro Manzano-Patron,
Paul S. Morgan, Stamatios N. Sotiropoulos


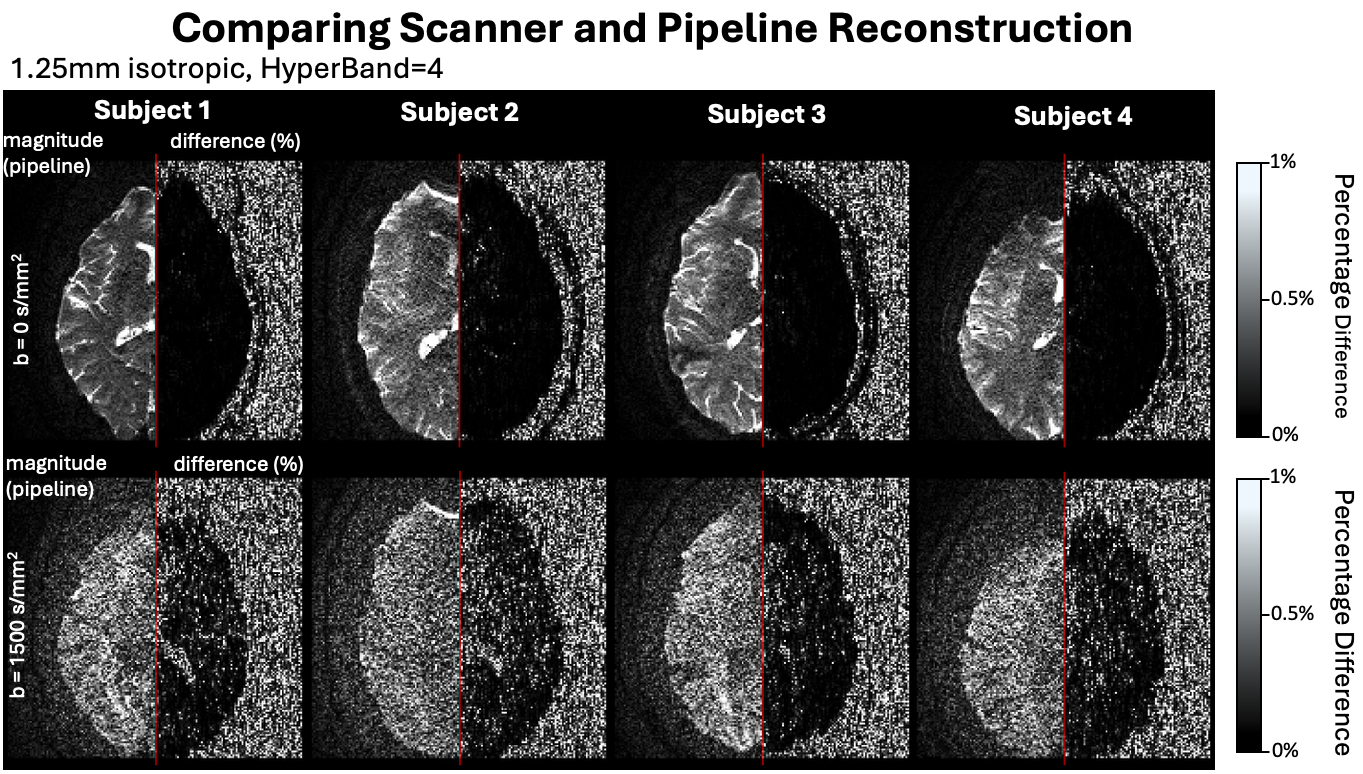


***Figure S1.*** *Validation of the on-scanner and offline pipeline reconstruction using experimental data from four subjects acquired for denoising comparison (1.25mm isotropic, Hyperband factor 4). Results are consistent with those shown in Figure 3, demonstrating high correspondence between reconstructions within the brain, with noisy differences mostly present outside the brain. Importantly, these results originate from a different site with a different software version (MR30.1) than those presented in Figure 3.*


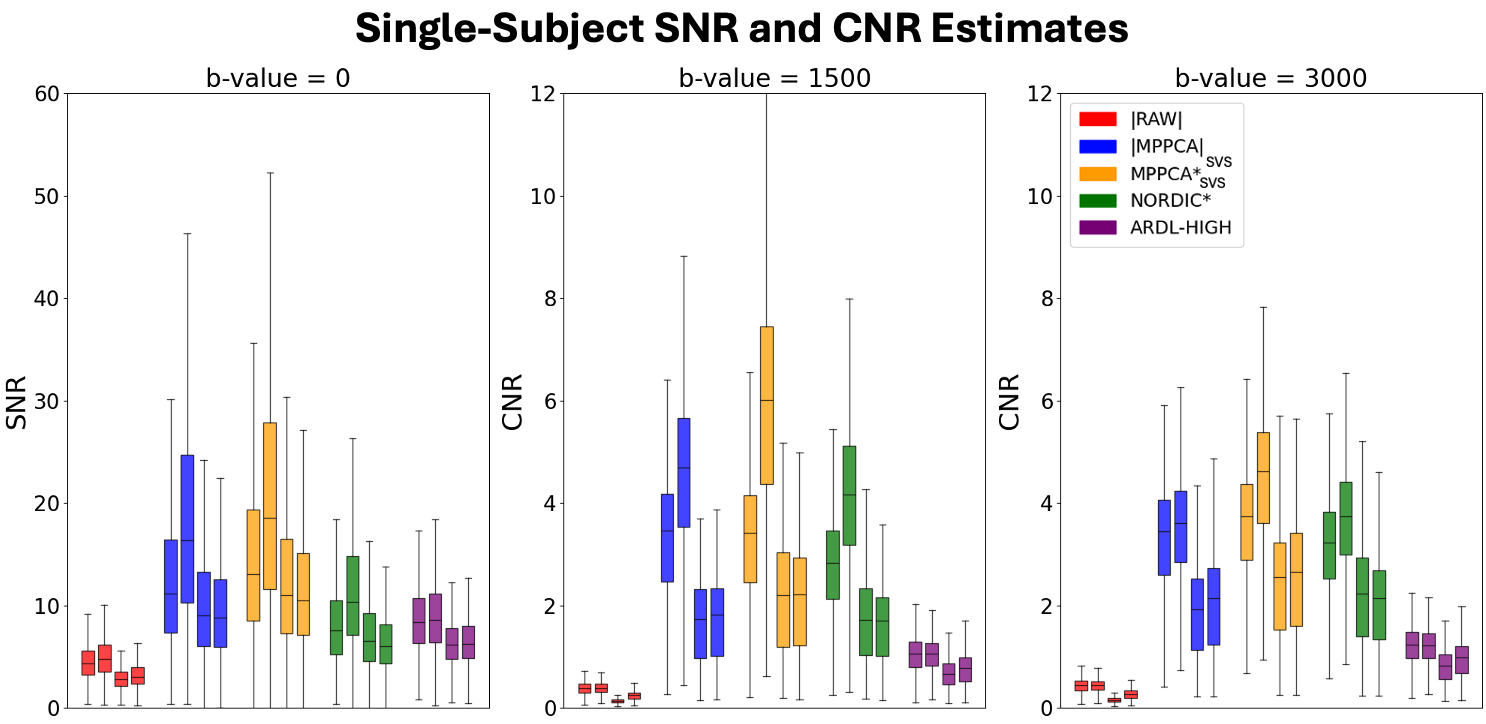


***Figure S2.*** *Single-subject SNR (across b=0 s/mm^2^ volumes) and angular CNR estimates using FSL-EddyQC.*

***
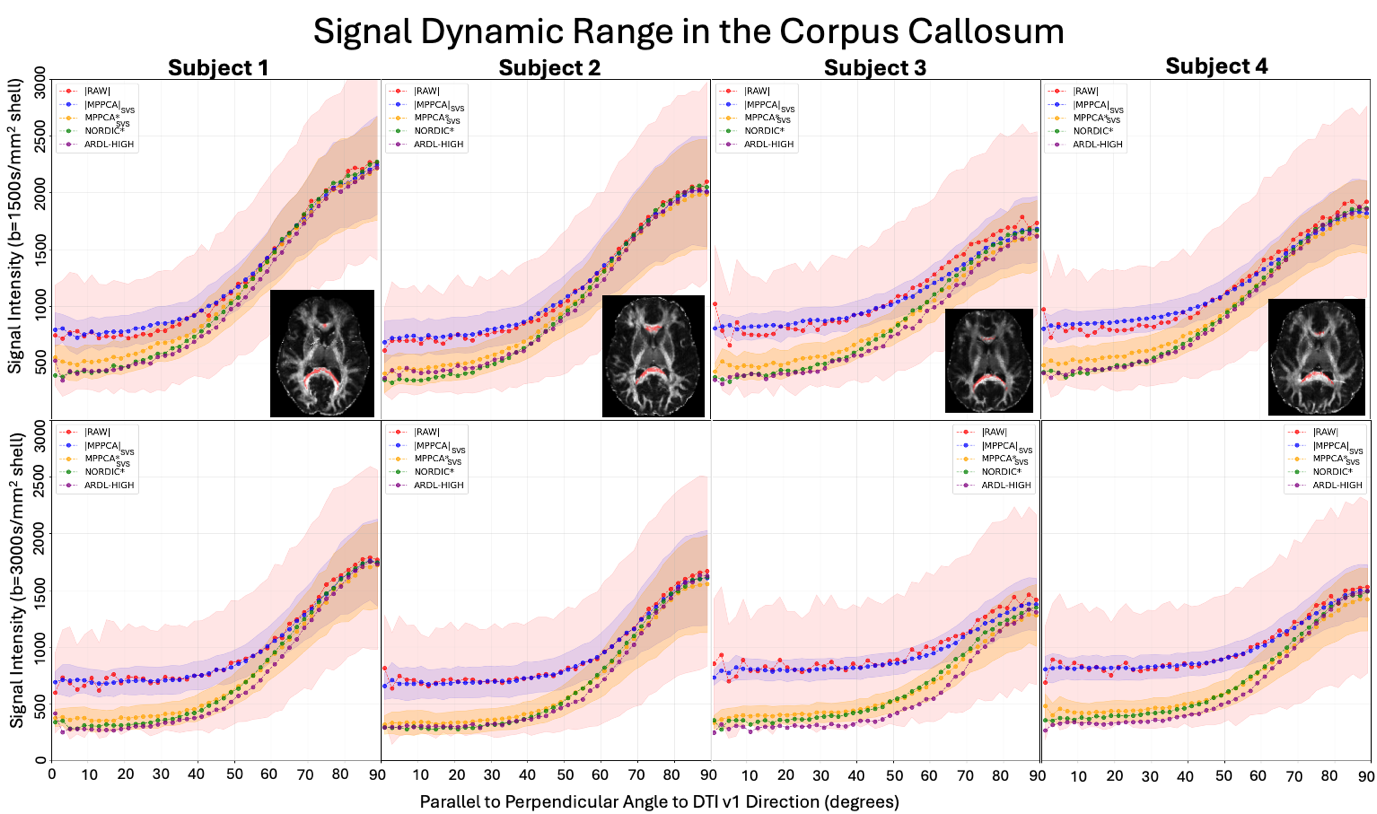
Figure S3.*** *Single-subject signal dynamic range in the Corpus Callosum (b=1500 s/mm^2^ – top, b=3000 s/mm^2^ – bottom).*


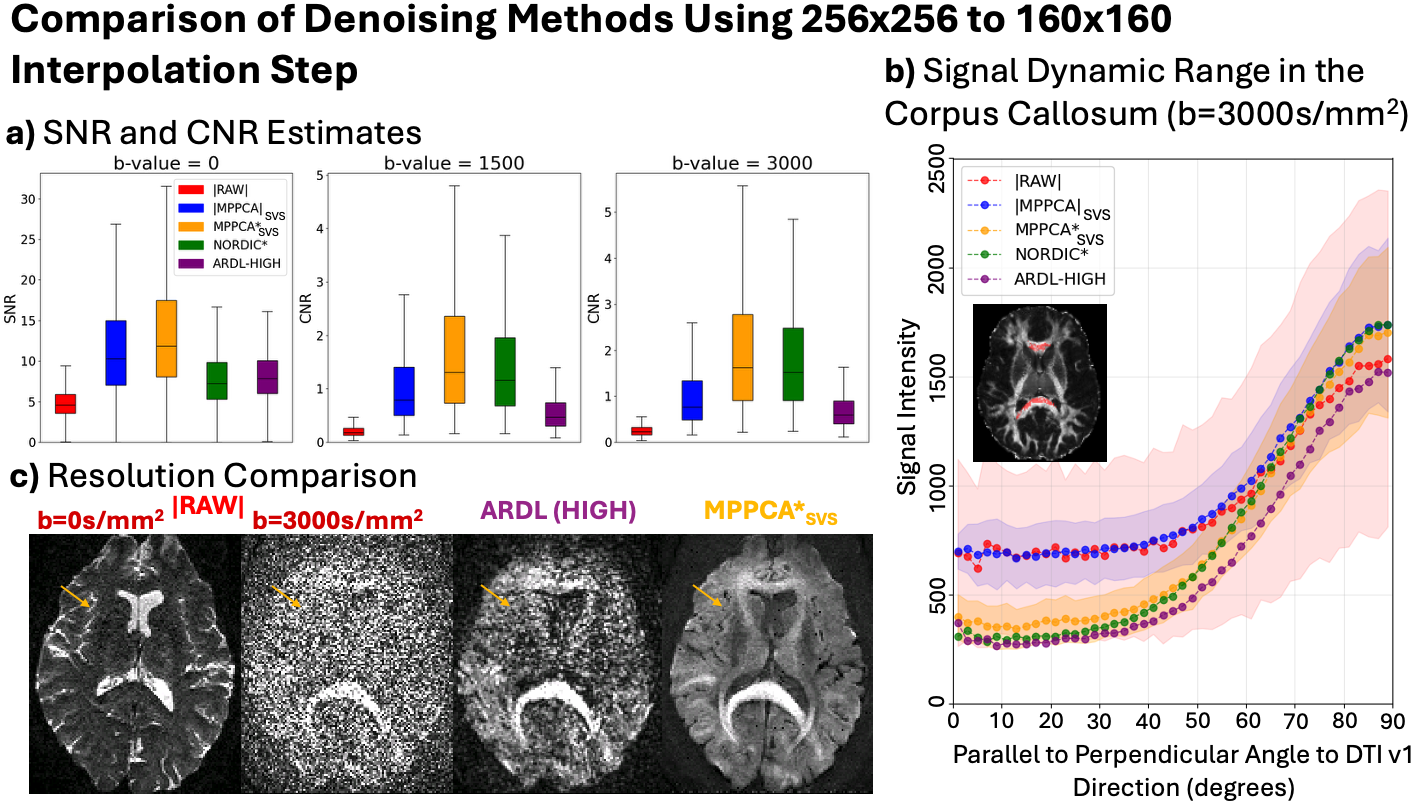


***Figure S4.*** *Comparison of denoising outcomes with identical interpolation steps across methods using a single subject. Denoising using patch-based approaches is carried out after the 256x256 to 160x160 step. a) SNR and CNR evaluation. b) Signal dynamic range in the corpus callosum normalized with respect to highest angle bin signal value. c) Comparison of resolution outcomes.*


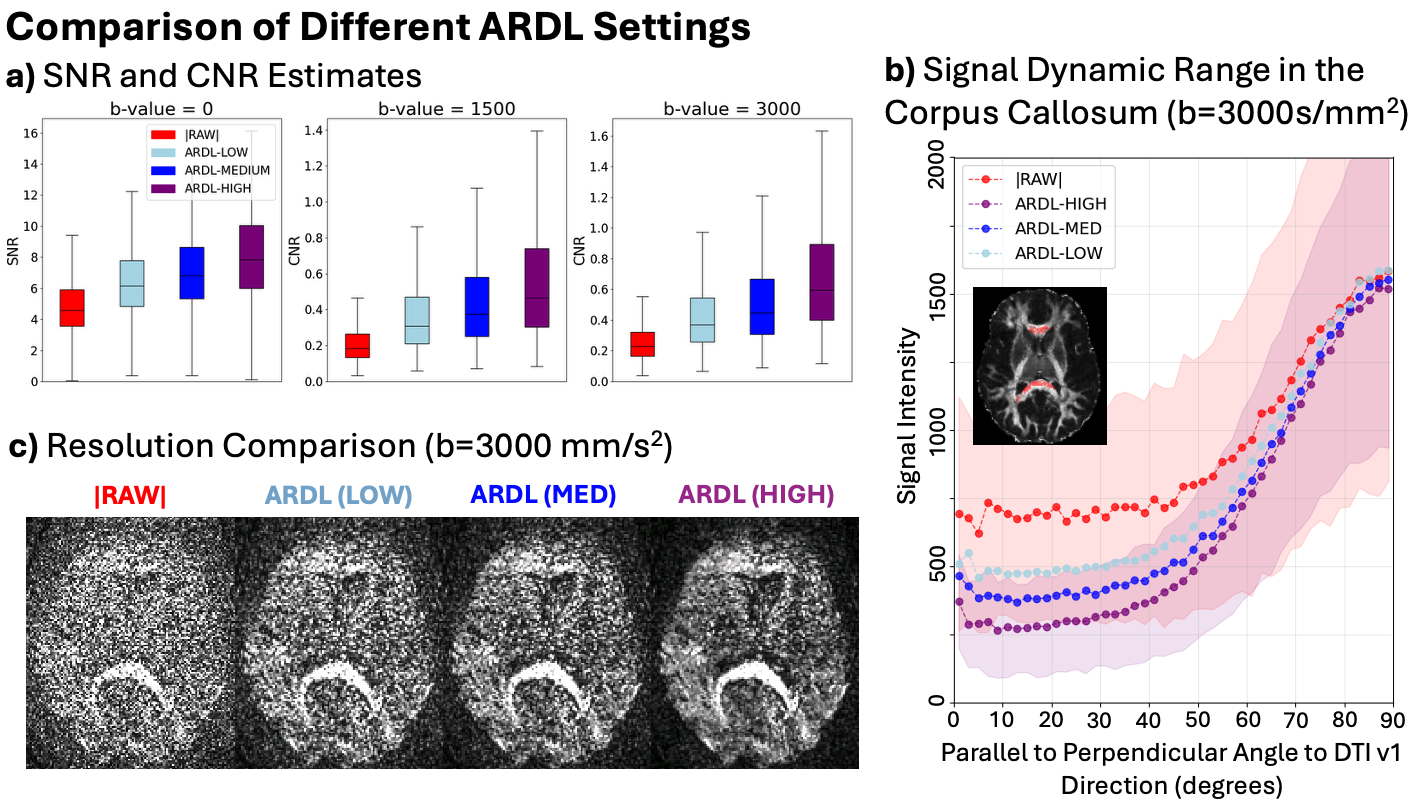


***Figure S5.*** *Comparison of denoising outcomes across ARDL settings (low – 0.3, medium – 0.5, high – 0.75) using a single subject. a) SNR and CNR evaluation. b) Signal dynamic range in the corpus callosum normalized with respect to highest angle bin signal value. c) Comparison of resolution outcomes.*

**
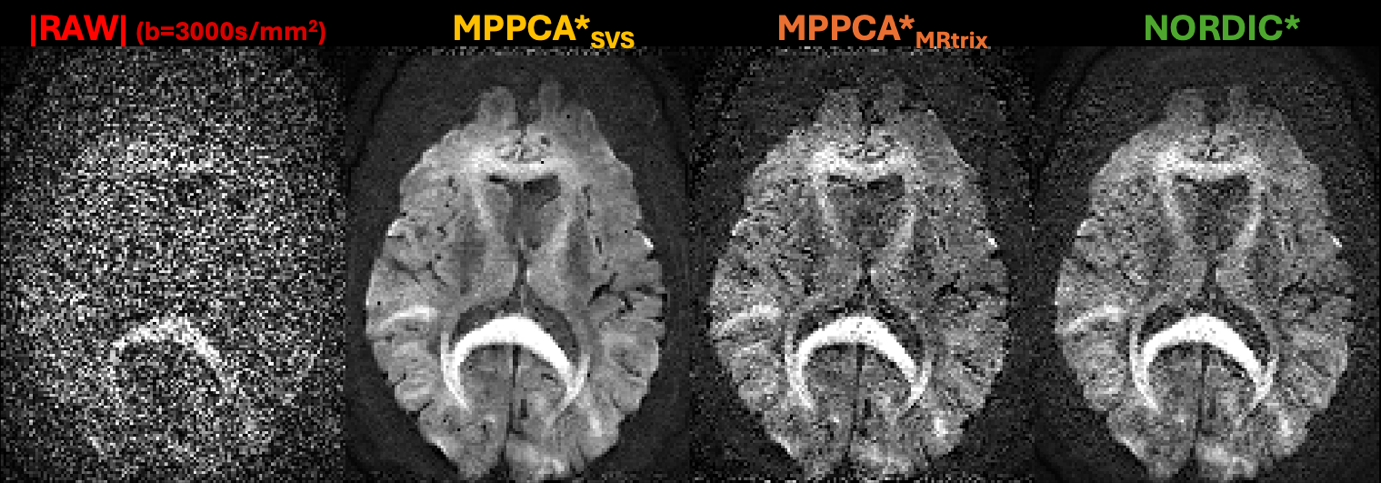
**

***Figure S6.*** *Qualitative comparison of |RAW|, MPPCA*_SVS_, MPPCA*_MRtrix_, and NORDIC* on a b=3000s/mm^2^ slice.*
